# Impact of physical exercise in cancer survivors during and after antineoplastic treatments

**DOI:** 10.18632/oncotarget.24456

**Published:** 2018-02-08

**Authors:** Martina Ferioli, Giorgio Zauli, Alberto M. Martelli, Marco Vitale, James A. McCubrey, Simona Ultimo, Silvano Capitani, Luca M. Neri

**Affiliations:** ^1^ Department of Morphology, Surgery and Experimental Medicine, University of Ferrara, Ferrara, Italy; ^2^ Department of Biomedical and Neuromotor Sciences, University of Bologna, Bologna, Italy; ^3^ Department of Medicine and Surgery, University of Parma, Parma, Italy; ^4^ CoreLab, Azienda Ospedaliero-Universitaria di Parma, Parma, Italy; ^5^ Department of Microbiology and Immunology, Brody School of Medicine, East Carolina University, Greenville, NC, USA

**Keywords:** cancer survivors, cancer treatments, psycho-physical symptoms, physical exercise, physical activity treatments

## Abstract

Cancer patients experience symptoms and adverse effects of treatments that may last even after the end of treatments. Exercise is a safe, non-pharmacological and cost-effective therapy that can provide several health benefits in cancer patient and survivors, reducing cancer symptoms and cancer treatment side effects. The purpose of this review is to describe how the physical exercise is capable to reduce cancer symptoms and cancer treatment side effects. We realized a pragmatic classification of symptoms, dividing them into physical, psychological and psycho-physical aspects. For each symptom we discuss causes, therapies, we analyse the effects of physical exercise and we summarize the most effective type of exercise to reduce the symptoms. This review also points out what are the difficulties that patients and survivors face during the practice of physical activity and provides some solutions to overcome these barriers.

Related to each specific cancer, it emerges that type, frequency and intensity of physical exercise could be prescribed and supervised as a therapeutic program, like it occurs for the type, dose and duration of a drug treatment.

## INTRODUCTION

Cancer symptoms and adverse effects of cancer therapies can be resolved or persist for days, weeks, or years. Physical activity is defined as any bodily movement produced by skeletal muscles that results in energy expenditure, which may be unstructured and everyday life activity, exercise that includes prearranged, deliberate and repetitive activity and grassroots sports and competitive sports [1].

The present review explores the impact of physical activity on physical, psycho-physical and psychological aspects on adverse effects of cancer. In particular the physical category includes bone loss and metastases, changes in body composition, cachexia, lymphedema and peripheral neuropathy; the psycho-physical category comprehends pain, fatigue and sleep disorders; the psychological category encompasses depression, anxiety, quality of life and self-esteem. For each aspect we report definition, causes (related to cancer or cancer treatment) and both pharmacologic and non-pharmacologic therapies. At the end of each paragraph we focus on the effect of physical exercise on specific symptoms and we analyse the most effective type of exercise to reduce the symptoms (if reported in the literature). We finally point out what are the barriers between patients/survivors and physical activity and how to overcome these difficulties.

## PHYSICAL ASPECTS

### Bone loss and bone diseases

Bone loss and diseases can be related to cancer metastasis or to cancer treatments, such as hormonal therapy for breast and prostate cancer patients.

Metastatic lesion can cause increased bone resorption (osteolytic lesions, typical of breast or prostate cancer), increased bone formation (osteoblastic lesions, typical of prostate cancer) or both mechanisms (mixed lesions). Bone metastases may cause severe pain, pathologic fractures, compression syndromes of the nerve root or of the spinal cord, metabolic disturbances (such as hypercalcemia and phosphate imbalances) and nephrolithiasis [2]. Bones are frequent sites of metastases of solid tumours: breast and prostate cancer patients have the highest prevalence of bone metastasis, followed by lung, gastrointestinal tract (colon and stomach) and genitourinary (bladder, kidney and uterus) cancer patients. Bone metastases are also frequently found in patients suffering from advanced thyroid cancer and melanoma. Multiple myeloma affects the bone marrow and consequently the bone in most of the cases (Table 1) [3].

**Table 1:** Examples of most frequent symptoms in some neoplasia

	Breast cancer	Gastrointestinal cancer	Gynaecologic cancer	Prostatic cancer	Head and neck malignancy	References
**PHYSICAL ASPECTS**						
**Bone Loss and disease**	✓	✓		✓		[3]
**Weight imbalance**	✓	✓	✓	✓	✓	[21, 22, 26, 28]
**Cachexia**		✓	✓	✓	✓	[49, 50]
**Peripheral neuropathy**	✓	✓				[72]
**Lymphedema**	✓		✓	✓	✓	[93–95]
**PSYCHO-PHYSICAL ASPECTS**						
**Pain**	✓	✓	✓	✓	✓	[108]
**Fatigue**	✓	✓	✓	✓	✓	[137]
**Sleep disorders**	✓		✓		✓	[160]
**PSYCHOLOGICAL ASPECTS**						
**Depression, anxiety**	✓		✓			[186]
**Quality of life and self esteem**	✓	✓	✓	✓	✓	[229, 230]

The decrease in bone mineral density that characterizes cancer treatment-induced bone loss often brings to osteopenia or osteoporosis, sometimes forcing drug suspension. Osteopenia can be distinguished from osteoporosis by measuring bone mineral density [4]. Cancer treatment can affect bone turnover by direct or indirect mechanisms. Hormonal therapies (such as long-acting gonadotropin-releasing hormone agonists and aromatase inhibitors) act directly on bone turnover by reducing circulating estrogen and testosterone levels. Some chemotherapeutic agents such as cyclophosphamide and doxorubicin induce hypogonadism. Estrogens and testosterone have an important role in regulating bone resorption, since estrogens increase osteoblast (OB) activity and proliferation and inhibit osteoclastogenesis, while testosterone inhibit OB apoptosis and promote OB proliferation. Some chemotherapeutic agents such as platinum derived agents and ifosfamide cause nephrotoxicity, resulting in alteration of the calcium regulation mechanisms through reduction of Vitamin D [5].

#### What are the possible therapies for bone metastasis and bone loss?

Bone loss in cancer patients has to be prevented and bone health has to be maintained by adopting life-style changes such as stop smoking, limit alcohol, supplement calcium intake and vitamin D, as well as take more weight-bearing exercise (see next paragraph). Bone metastasis requires a multidisciplinary management that includes external beam radiotherapy or radioisotopes therapy and bone targeted agents. Bone targeted agents include bisphosphonates (such as etidronate and alendronate) and denosumab. Bisphosphonates accumulate in the skeleton, reduce bone resorption, increase mineralisation by inhibiting osteoclast activity and are used also for widespread bone pain or pain recurrence after radiotherapy. Denosumab is a monoclonal antibody that binds Receptor Activator of Nuclear factor Kappa-Β Ligand (RANKL) preventing the binding between RANKL and RANK (Receptor Activator of Nuclear factor Kappa-Β) that lead to osteoclasts activation. Data suggest that the administration of bisphosphonates or denosumab could prevent the formation of breast and prostate cancer bone metastases [6] and therefore of cancer treatment-induced osteoporosis [7, 8].

#### Positive effect of physical activity on bone

There is a consistent body of evidence that exercise has a crucial impact on bone health. The effect of exercise on bone mass is all life-long: exercise prior to the pubertal growth stimulates greater accumulation of peak bone mass, in adults exercise preserves and may stimulate increase in bone mass density and physical activity is capable to reduce bone loss in the elderly [9]. A meta-analysis of 19 randomized control trials reported that exercise produced a significant improvement in lumbar spine and femoral neck bone mass density in older adults [10]. A recent systematic review focused on the judo effects on bones found that the balance and strength requirements of this sport generate osteogenic stimuli, causing increases in bone mass density of adolescents, adults and in pre- and post-menopausal women [11].

Impact and weight exercise training has to be preferred for bone health. Impact exercises include physical activities such as running and jumping that causes mechanical loads on bones, stimulating bone formation and promoting structural modifications of the bone tissue. Weight training involves muscle pull and muscular insertion of the skeletal sites of interest producing a delay in the loss of bone mineral density in pre and post-menopausal women with breast cancer [12].

Several studies on physical exercise in cancer patients evidenced a positive effect of impact plus resistance training on bone mass density. High-impact training (such as running and jumping) combined with high-resistance activities are effective in increasing bone mineral density in premenopausal women at the hip and spine level [13]. Exercise programs that mixed impact exercise (such as jogging) with low-impact activity (such as walking) or training that combined impact exercise (such as jumping) with resistance exercises are effective in preserving bone mineral density in postmenopausal women [14].

Combined training programs of impact and resistance improved bone mass density in the hip and prevented bone mass density loss in the spine in breast cancer survivors with premature treatment-induced menopause [15], while seems to prevent only the bone mass density loss in the spine and not in the hip in postmenopausal breast cancer survivors (Table 2) [16]. In fact, this different response of exercise on total hip bone mass density is influenced by age: in particular the effect of impact and resistance exercise on hip within postmenopausal breast cancer survivors diminishes with age [17].

**Table 2:** Physical exercises and their reduction of cancer-related symptoms

	Aerobic Exercise	Resistance exercise	Strength exercise	Weight and Impact Exercise	Balance Exercise	Flexibility and Relaxation Exercise	References
**PHYSICAL ASPECTS**							
**Bone Loss and disease**		✓		✓			[11, 15]
**Muscle and fat mass imbalance**	✓		✓				[34, 35, 38]
**Cachexia**	✓	✓					[59, 60, 64]
**Peripheral neuropathy**			✓		✓		[91, 92]
**Lymphedema**		✓					[99]
**PSYCHO-PHYSICAL ASPECTS**							
**Pain**	✓						[132]
**Fatigue**	✓	✓	✓			✓	[135, 151]
**Sleep disorders**	✓					✓	[163, 181]
**PSYCHOLOGICAL ASPECTS**							
**Depression, anxiety**	✓						[217]
**Quality of life**	✓	✓	✓			✓	[230, 232]
**Self Esteem**		✓					[237]

A recent trial involving female cancer survivors found that also aerobic training when combined with resistance-training exercise produced a significant increase in bone mass density at the spine, hip and whole body. Aerobic exercise consisted in walking or using the elliptical machines, while resistance-training included upper and lower body resistance exercises provided through use of body weight, dumbbells, elastic bands [18]. Physical exercise may also play an important role in reducing bone loss and preventing fracture risk in prostate cancer patients treated with androgen suppression treatment [19].

### Muscle loss and weight imbalance

Cancer patients experience muscular and weight changes due to the cancer itself or to the cancer treatments, such as loss of muscular mass, increased fat mass and weight gain. Increased fat mass and obesity are significantly associated with increased cardio-metabolic disease risk that lead to an augmented mortality of cancer survivors, while reduction in muscular mass could promote the development of cancer syndromes such as cachexia, making important to assess their presence and to prevent worsening due to these changes.

Muscular changes in cancer patients include significant impairments in muscle strength and loss of muscle mass, related to both cancer itself and cancer treatments.

Causes of reduced muscle mass induced by cancer include malnutrition, physical inactivity, altered metabolism and increased proinflammatory cytokines such as Tumor Necrosis Factor- α (TNF-α), Interleukin-1-β (IL1-β) and Interferon-γ (IFN-γ), that seem to upregolate muscle degradation as detected in muscular biopsies of cancer patient.

Cancer therapies may affect muscle mass with different mechanisms: chemotherapy has a cytotoxic and an anti-proliferative effect, but also cause nausea, diarrhea and anorexia, which worsen the nutritional intake, while surgery and/or radiotherapy may destroy muscle structures in the treated areas [20]. Loss of muscle mass during chemotherapy is associated with poor survival and it has been reported in patients with ovarian [21], respiratory, gastrointestinal [22], melanoma [23] and metastatic colorectal cancers (Table 1) [24]. Loss of muscle mass often brings initially to precachexia, characterized by weight loss (less than 5%), anorexia and metabolic changes (such as impaired glucose tolerance) and then to cachexia [25].

Loss of muscle mass may be masked by the increase in fat mass and the weight gain associated with the cancer therapy such as chemotherapy, steroid medication and hormonal therapy. Increase in fat mass is a typical side effect of androgen deprivation therapy in prostate cancer patients. The increase in fat mass usually induced by the androgen deprivation therapy predispose prostate cancer patients to develop cardiovascular diseases and it’s also associated with cancer recurrence and higher specific prostate cancer mortality [26]. Cisplatin-based chemotherapy may cause an increase in abdominal visceral and subcutaneous adipose tissue [27].

Weight gain can be caused by chemotherapy, steroid medication and hormonal therapy. Weight gain is common in breast cancer patient receiving chemotherapy and survivors given that incidence is ranging from 50% to 96%, with 20% of them increasing more than 10 kg [28]. Weight gain results as a combination of several factors such as chemotherapy (especially the combination of cyclophosphamide, methotrexate and 5-fluorouracil) [29], decreased physical activity, menopause and changes in basal metabolic rate [30]. Weight gain has effect on psychosocial aspect of life, such as body image, as well as on physical problems like the aforementioned cardiovascular and metabolic diseases (such as diabetes mellitus) [31]. A meta-analysis reported that weight gain (especially greater than 10%) after the diagnosis of breast cancer is a negative prognostic factor associated with increased all-cause mortality rates when compared with body weight manteinance [32].

#### What are the possible therapies for muscle loss and weight imbalance?

Weight gain and increase of fat mass can be controlled by following a diet (that has to prefer fruits, vegetables, and whole grains and to limit fat, sugar, and refined flour) and by exercise (see next paragraph). The reduction of muscle mass may bring to cancer cachexia, so it’s important to recognize its loss and start as soon as possible preventing the development of precachexia and cachexia [33].

#### Positive effect of physical activity on muscle and fat mass

Exercise is one of the main treatment that can act on muscle strength, fat mass and therefore weight gain.

Either resistance or strength training cause an increased synthesis of actin and myosin, resulting in reduced muscle weakness, augmented muscle mass and thus muscle strength [34]. In breast cancer survivors a strength training program (performed twice a week, for 6 to 12 month) determines significant changes in body composition by decreasing percentage of body fat and increasing lean body mass [35] and causes improvements of upper and lower body strength (Table 2) [36]. A study on breast cancer patients performing a combined aerobic and resistance training (three times per week for 16 week) reported a significant increase in leg and trunk strength [37].

Similarly, in breast cancer patients undergoing chemotherapy, a single aerobic exercise, such as a walking training program (performed 5 consecutive days per week, for 12 weeks), caused a significant weight and body fat percent reduction [38]. In prostate cancer patient receiving androgen deprivation therapy, a 3-month exercise programme (performed twice a week) that combined exercise and resistance training preserved appendicular lean mass decay and prevented gains in whole body fat mass [39].

Several studies found positive results of physical exercise in prostate cancer patient that received androgen deprivation therapy, suggesting that exercise should become an important part of clinical care in these patients. In particular, 12-month of impact plus resistance exercise program can increase maximal leg strength [40].

Physical exercise is not only capable of reducing fat mass, but also of lowering the consequences of fat mass increase, such as greater risk of cardiac and metabolic disease. In particular, exercise causes cardiorespiratory changes (such as lowering blood pressure) and metabolic changes (such as improving insulin sensitivity, reducing blood lipid levels and enhancing glycemic control) that can prevent cardio-metabolic cancer comorbidities [41].

### Cachexia

Cancer cachexia is a paraneoplastic syndrome characterised by a progressive loss of skeletal muscle mass (with or without loss of fat mass) that cannot be completely reversed by conventional nutritional support and leads to progressive functional impairment. Approximately 20% of cancer deaths are directly caused by cachexia [42].

The diagnostic criteria for cancer cachexia are: a weight loss >5% over past 6 months (in absence of simple starvation) or a BMI <20 and ongoing weight loss of more than 2% or appendicular skeletal muscle index consistent with sarcopenia (males <7,26 kg/m^2^; females <5,45 kg/m^2^) concomitant with any degree of weight loss >2% [25].

The origin of cancer cachexia is multifactorial and includes reduction of food intake and abnormal energetic metabolism, driven by proinflammatory cytokines released by tumour cells, such as IFN-γ, TNF-α and nuclear factor kappa-light-chain-enhancer of activated B cells (NF-kB) [43].

Concerning the lipid metabolism, interleukin-1 (IL-1) and IFN-γ have been related to stimulation of lipolysis and TNF-α is a potent inhibitor of lipogenesis, since it decreases lipoprotein lipase (LPL) activity and reduces the synthesis of transcription factor essential for the adipocyte differentiation [44].

The most important changes of carbohydrate metabolism are: glucose intolerance, insulin resistance and increased hepatic gluconeogenesis. Gluconeogenesis is promoted by the high level of lactate derived from tumour glycolysis and is insensitive to physiological inhibitor stimuli such as the administration of glucose itself. Gluconeogenesis is an extremely energy-demanding process [45] and may be considered an important mechanism of the increased energy consumption in cancer patients. Gluconeogenesis also influences the protein metabolism, recruiting and therefore reducing amino acid levels.

A central alteration of the protein metabolism in cancer cachexia is the reduction of lean body mass. The depletion of lean mass is caused by an increase of muscular protein degradation without a correspondent increase of protein synthesis, which remains normal or is even slightly reduced [46]. There are three main mechanisms of muscle proteolysis: the lysosomal (cathepsin) proteolytic pathway, the calcium-activated system and the ubiquitin-proteasoma proteolytic pathway, which is the most important mechanism of protein degradation in cachexia [47]. One of the main mediators of this mechanism is the proteolysis-inducing factor (PIF), but also TNF-α and IL-1 play an important role [43] and feeding-regulatory peptides such as leptin, ghrelin, and melanocortin are involved [48].

#### What kind of cancer/therapy can cause cachexia?

Approximately 50% of all patients with cancer experience cachexia. The prevalence of cachexia is highest in patients with pancreatic (about 85%), gastric and esophageal cancer while urological (8%), gynaecological (15%) breast and lymphoma cancer patients are less affected (Table 1) [49, 50].

Not only cancer disease but also antineoplastic treatments influence cachexia and interfere with the nutritional state maintenance. Both chemotherapy and radiotherapy cause side effects such as nausea, vomiting, swallowing difficulties, mucositis, taste change and fatigue and worse the nutritional imbalance also through the development of systemic inflammation [51].

#### What are possible treatments for cachexia?

Since weight loss may continue for about a year after cancer removal, it is clear that the goal treatment for cachexia is the reversal of the loss of body weight and muscle mass [42]. The treatment approach has to be multimodal and includes nutritional therapies to increase protein intake, pharmacological therapies to reduce inflammation and improve nutritional balance and not less important, exercise therapies.

The pharmacologic approach for cachexia includes different drugs, such as steroid hormones and cyclooxygenase inhibitors. Steroid hormones (especially corticosteroids and progestins) are used to stimulate appetite but they are associated with increase of fat mass, water retention and thromboembolism risk. Cyclooxygenase inhibitors (such as indomethacin and ibuprofen) reduce the inflammatory status. Thalidomide reduces inflammation inhibiting the production of TNF-α, but this drug is teratogenic and may cause severe adverse effects such as pulmonary oedema, hypotension and renal insufficiency [33].

However, lot of trials are assessing the potential clinical benefit of new agents involved in appetite stimulation, anti-inflammatory pathways, anabolic or catabolic pathways but the description of these agents is out of the purpose of this review. The non-pharmacologic strategies to fight cachexia include diet modification and physical activity.

#### Positive effect of physical activity on cachexia

Exercise has a potential role in preventing and treating cachexia [52]. In fact, exercise influences inflammation and metabolism, the two main mechanisms of cancer cachexia.

Exercise influences inflammation by lowering the release of proinflammatory cytokines such as TNF-α [53] and promoting the production of mediators with anti-inflammatory effects, especially myokines, such as interleukin-6 (IL-6). IL-6 improves insulin sensitivity and results in an increase in the concentrations of IL-1ra (interleukin-1 receptor antagonist) and IL-10, two anti-inflammatory cytokines and stimulates skeletal muscle oxidative metabolism [49, 54]. Exercise also has an antioxidant effects by activating oxidative damage repairing enzymes [55].

Exercise has an important role on carbohydrate metabolism, since carbohydrates are the major sources of energy during moderate and high-intensity exercise [56], and furthermore is able to increase muscle glucose uptake and insulin sensitivity also in patients with cachexia when suffer of glucose intolerance and insulin resistance [57].

Exercise is an approach to enhance muscle protein synthesis, especially if resistance-based exercise is combined with provision of branched chain amino acids [58].

Exercise can also reduce muscular atrophy by recruiting and activating muscle satellite cell and stimulating muscle protein synthesis. Muscle satellite cells can re-enter the cell cycle and multiply, contrasting the loss of muscle mass and promoting muscle regeneration. Up to date, the effect of cancer treatment on activated satellite cells is unknown [52].

Which kind of physical exercise could reduce cachexia? Several studies tested different programs of exercise. Progressive resistance training (PRT) is associated with increase of muscle mass and bone mineralisation in both healthy people and elderly sarcopenic individuals (Table 2) [59]. To assess this improvement also in cachectic patients, Lønbro et al. conducted a trial in which head and neck cancer patients performed a resistance-training program (2-3 sets of repeated 8-15 times of seven conventional exercise, including leg press, knee extension, hamstring curls, chest press, sit ups, back extensions and lateral pull down). The results showed an increase in lean body mass [60]. Possible mechanism to explain the positive benefits of resistance training may be the increase of insulin like growth factor 1(IGF-1) and the activation of proliferator-activated receptor gamma coactivator 1 alpha (PGC-1α) [61]. In fact, IGF-1 is involved in the hypertrophy signaling of skeletal muscles [62], while PGC-1α is a transcriptional coactivator that regulates the expression of genes involved in oxidative metabolism. The expression of PGC-1α is enhanced during resistance exercise and is associated with fiber-type switching, stimulation of fatty acid oxidation, angiogenesis and resistance to muscle atrophy. Furthermore, a specific form of PGC-1α (PGC-1α4) is probably involved in in skeletal muscle hypertrophy [63].

Several studies conducted in Walker 256 tumour-bearing rats (a model that simulates many of the alterations caused by human tumours) reported that aerobic exercise is associated with a retarded skeletal muscle degradation and a reduction of body mass loss [64].

Given the possible effects of both resistance and aerobic exercises, a combination of these trainings has been recently proposed to treat cachexia. However, definitive regimens have to be further developed to support the efficacy and safety of exercise training for cancer cachexia patients [65].

### Peripheral neuropathy

Peripheral neuropathy is a condition that results from peripheral nerves damage. Peripheral neuropathy can occur as a paraneoplastic manifestation, but in most of the cases it is a side effect of anti-cancer treatment, and therefore is called chemotherapy-induced peripheral neuropathy (CIPN).

The supposed pathogenesis of CIPN is due to the neurotoxic effects of chemotherapy and the lack of protection of the peripheral nervous system. The cell bodies of the dorsal root ganglia are particularly vulnerable to neurotoxic damage because the blood–brain barrier doesn’t protect them; furthermore they are supplied by fenestrated capillaries that make them more accessible to neurotoxic circulating agents. The neurotoxic effects of chemotherapy are probably related to the drug mechanisms of cytotoxicity in cancer cells, so the neurotoxicity depends on either the agent used and the cumulative dose [66].

Peripheral neuropathy consists commonly in a pure sensory neuropathy, with sensory symptoms (such as tingling, hyperalgesia and allodynia), but sometimes it can cause motor or autonomic symptoms (such as hypotension, constipation and erectile dysfunction). Patients suffering from CIPN usually experience firstly numbness and loss of deep tendon ankle reflexes and then paresthesia and loss of positional sense [67].

CIPN prevalence is estimated to be about 68% in the first month after chemotherapy, 60% at 3 months and at 6 months 30% of patients continue suffering from CIPN [68]. CIPN may persist for years in cancer survivors [69] determining a significant disability and a decreased quality of life.

CIPN influences the course of disease and treatment: sometimes it’s necessary to lower the chemotherapy dose, delay or even stop the cancer treatment [70]. CIPN seriously impact quality of life of the patient, reducing mobility and limiting their ability to perform simple daily activities [71].

#### Which chemotherapeutic agents can cause CIPN?

Chemotherapeutics that causes CIPN include platinum drugs (such as cisplatin, carboplatin and oxaliplatin), Microtubule-Targeting Agents (- MTAs - such as taxanes and vinca alkaloids), proteasome inhibitors (such as bortezomib) and angiogenesis inhibitors (such as bevacizumab and thalidomide) [67].

Platinum chemotherapies, commonly used for the treatment of solid tumours (e.g. breast, colon, lung and testicular cancers), determine a sensory peripheral neuropathy (with numbness, tingling and paresthesia, decreased vibratory sensation and loss of proprioception) and often cause worsening for several months after the end of treatments, a phenomenon called “coasting” (Table 1) [72]. Platinum chemotherapies act by forming Platinum–DNA adducts that finally cause the apoptosis of cancer cells, but unfortunately also of sensory neurons, in which platinum chemotherapies determine morphological alterations in the nucleolus, mitochondrial dysfunctions and increase production of reactive oxygen species [73].

Taxanes (such as paclitaxel and docetaxel) cause a sensory neuropathy that typically includes paresthesia, numbness or neuropathic pain in hands and/or feet. Symptoms often improve within 3-6 month after the end of the treatment, but sometimes the neuropathy doesn’t resolve [74].

Vinca alkaloids (used to treat acute lymphocytic leukemia, lymphomas, and neuroblastoma) alter the axonal cytoskeleton (length and orientation of microtubules) inducing a sensory neuropathy similar to taxanes but are also associated with an autonomic neuropathy [75].

Although the neurotoxicity mechanisms of thalidomide are unknown, CIPN is one of the most common side effects of this drug, a glutamic acid derivative used for the treatment of the multiple myeloma, and symptoms are often both sensorial and autonomic [76].

Bortezomib is a proteasome inhibitor used for multiple myeloma that causes chronic sharp sensation, burning pain, and sometimes autonomic neuropathy. The neurotoxic action of bortezomib is probably due to tubulin polymerization, mitochondrial changes, oxidative stress and increased production of glutamate [66].

Bevacizumab (an angiogenesis inhibitors that acts on vascular endothelial growth factor, VEGF) when administered in combination with oxaliplatin, can enhance peripheral neuropathy induced by the platinum agents [77].

#### What are the possible strategies to prevent and treat CIPN?

Many agents have been tested for the CIPN prevention, such as acetyl-L-carnitine, amifostine, amitriptyline, glutathione, nimodipine and vitamin E, with different results. Anyway, the American Society of Clinical Oncology (ASCO) Clinical Practice Guidelines don’t recommend any agents for the prevention of CIPN in cancer patients undergoing neurotoxic agents, because there aren’t high-quality evidences for benefits derived from these agents [78].

ASCO recommend duloxetine, a serotonin-norepinephrine reuptake inhibitor (SNRI) antidepressant with positive results for the treatment of CIPN [79]. Topical agents that include lidocaine, baclofen, amitriptyline and ketamine are commonly used for the treatment of CIPN, thanks to the localized anesthetic properties. The relief is rapid but with short duration, so the agent has to be reapplied frequently [80]. Other drugs used for the treatment of CIPN are venlafaxine (another SNRI), tricyclic antidepressants (such as nortriptyline/amitriptyline) and antiepileptic agents (such as gabapentin and pregabalin) commonly used for the management of neuropathic pain syndromes. Alpha-lipoic acid (ALA) and acetyl-L-Carnitine (ALC) appear to be effective for the treatment of diabetic neuropathy, while more data are needed to evaluate their efficacy for the management of CIPN. Other emerging therapies include topical menthol and capsaicin, used as analgesic agents [81].

A multifunctional approach for the treatment of CIPN includes also nonpharmacologic interventions, such as acupuncture [82], scrambler therapy (a cutaneous electro-stimulation to reduce pain sensation of CIPN) [83] and exercise.

#### Positive effect of physical activity on peripheral neuropathy

Despite the widespread belief that physical activity is not safe for persons with peripheral neuropathy because of the augmented risk of falling, Kruse et al. reported that exercise is feasible and safe for diabetic patients (which have an high risk of foot ulceration and fall) with peripheral neuropathy [84].

Also cancer patients and survivors suffering from CIPN are at risk of postural instability, falls and fall related injury, but data suggest that exercise is a safe intervention for these patients [85].

Exercise has a positive role on peripheral neuropathy in mouse receiving paclitaxel. A study that examined the effect of a rigorous treadmill exercise program, started 1 week before administration of the drug and continued during the therapy, found that exercise could partially reduce axonal degeneration. In particular they found that mouse which exercised did not show a reduction in epidermal nerve fiber density, did not suffer from thermal hypoalgesia and did not have an elevated detyrosinated tubulin levels, features typically associated with paclitaxel administration [86].

Also clinical trial conducted on patients investigated the role of exercise in the management of peripheral neuropathy [71]. Two studies that tested a structured 10-12 weeks exercise programs (one supervised, the other home-based) found that exercise training reduces CIPN symptoms, such as unpleasant skin sensations, sensitivity related to neuropathic pain and the experience of coming on suddenly in bursts for no apparent reason [87, 88].

How exercise may work on nerves? This question has only a partial answer, because the mechanism is complex and is not clear. Exercise contrasts the progressive muscle mass atrophy associated with neuropathy by increasing muscular strength. Exercise is also associated with improved muscle reinnervation and increased axon regeneration [89]. Probably neurotrophic factors such as brain derived neurotrophic factor (BDNF) and glial cell-derived neurotrophic factor (GDNF) are involved in the peripheral nerve regeneration promoted by exercise [86, 90].

In patients with peripheral neuropathy, some studies tried to define the most effective type of training, examining strength and balance training. Balance training has the aim to improve stability and prevent falls by strengthen legs and core muscles. Balance exercises include stance on heel/toes, tandem stance, one leg stance and different kinds of walking. Allet et al. reported that a strength and balance training program (60 minutes, twice a week for 12 weeks) is able to increase walking speed, improve balance and strength and reduce fear of falling in patients with peripheral neuropathy [91]. A systematic review done by Streckmann et al. that included 18 studies referred to patients with peripheral neuropathy (due to diabetes, CIPN or other reasons) found that balance training seemed to be the most effective exercise intervention compared to endurance or strength training program (Table 2) [92].

In summary, exercise (especially strength and balance training) is a potentially effective preventive and therapeutic approach to contrast and reduce the neurotoxic effects of chemotherapy (for example of platinum chemotherapies, commonly used for the treatment of colon cancer) and positively impacts the lives of patient with CIPN. The mechanisms is not completely clear but probably involves BDNF and GDNF, that promote muscle reinnervation and axon regeneration.

### Lymphedema

Lymphedema is an abnormal collection of protein-rich interstitial fluid (lymphatic fluid) that results from lymphatic insufficiency and inadequate lymph transport. Lymphedema is usually classified as primary or secondary. The primary lymphedema has a congenital origin (absence or malformation of lymphatic vessels). The secondary lymphedema has an infective or iatrogenic origin, which can be surgical (because of the removal or damage of lymph nodes) or related to radiation therapy because of the lymph nodes fibrosis. Lymphedema can cause complications such as delayed wound healing, skin break-down, infections, lymphangitis and lymphangiosarcoma, a rare form of lymphatic cancer. Lymphedema can also influence daily activities by causing a restriction in range of motion, pain, increased skin tension and a sense of heaviness of the affected limb. Impairment of function reduces the ability to work and decreases quality of life [93].

#### What types of cancer/therapy can cause lymphedema?

The overall risk of lymphedema for all cancers is estimated to be 15.5% [94]. The risk of developing lymphedema is a lifelong risk that doesn’t diminish over time (Table 1) [95]. Lymphedema may occur after the surgical treatment of breast cancer, melanoma, gynaecologic and prostatic cancer, head and neck malignancies and sarcoma.

Upper extremities lymphedema is a long-term complication of axillary lymph node dissection or even of sentinel lymph node biopsy alone. The degree of lymphedema depends on the number of lymph nodes removed and the extent of radiation treatment to the axillary region. Most of breast cancer survivors develop lymphedema within three years from the treatment [93].

Lower Limb Lymphedema is the most frequent expression of lower body lymphedema and is generally observed in patients treated for gynaecologic malignancy or prostate cancer. Incidence and timing of secondary lower body lymphedema is variable depending on the study because there isn’t a uniform diagnostic definition [96].

#### What are the possible treatments for lymphedema?

The management of lymphedema consists in surgical or non-surgical (conservative) therapy. The conservative treatment is referred to complex decongestive therapy, which includes manual lymphatic drainage, compression therapy and exercise (see below).

Manual lymphatic drainage consist in slow, very light repetitive circular massages done in a specific sequence to facilitate the removal of retained interstitial proteins, redirect fluid to lymphatic vessels, stimulate lymph flow and promote the development of accessory lymph pathways [97].

Manual lymphatic drainage is used in combination with compression therapy, which consists in setting low-stretch multi-layered bandages to improve fluid reabsorption and enhance lymphatic contraction and flow [93].

The surgical therapy is a secondary option indicated in case of fail or strict adherence to complex decongestive therapy [97]. Medications such as diuretics are not indicated in the long-term treatment of lymphedema [96] and only in case of acute cellulitis the use of antibiotics is indicated. Skin care and education are important to prevent the risk of lymphedema [97].

#### Positive effect of physical activity on lymphedema

Physical exercise can be both a treatment and a prevention strategy for lymphedema.

A BMI higher than 30 kg/m^2^ is a significant risk factor for lymphedema, so physical activity is important to prevent this condition [52].

The American National Lymphedema Network (NLN) confirms that a safe form of exercise is an essential part of a fitness program for people with lymphedema and defines five types of exercises: lymphedema remedial exercise, flexibility or stretching exercises, cardiopulmonary exercises, resistance or weight-lifting exercises and combined resistance and aerobic exercises. The lymphedema remedial exercise consists in the repetition of active non-resistive movement of the involved body part and therefore in the use of the natural muscle pump to increase venous and lymphatic fluid flows from the swollen areas. This kind of exercise is an integral part of the complex decongestive therapy and should be adapted to the patient’s comorbidities and level of activity [97, 98].

Flexibility or stretching exercises improve the lymphatic function by preserving the range of motion and reducing fibrous adhesions due to surgery or radiation. Cardiopulmonary exercises such as walking, cycling and swimming have not been studied formally as a treatment for lymphedema. Resistance or weight-lifting trainer programs are currently the most studied exercises for breast cancer survivors. Even if it is a widespread opinion that breast cancer survivors should avoid upper body exercise, a progressive resistance exercise training does not increase the incidence of lymphedema and conversely this kind of exercise may determinate favourable effects in women that are at risk (Table 2) [99] clearly decreasing the signs and symptoms of lymphedema [100].

A recent systematic review of six articles (involving 805 breast cancer survivors) assessed the efficacy of resistance exercise among patient with or at risk for lymphedema [101]. This review also elaborates preliminarily recommendations regarding these exercises. The resistance exercise program should be prescribed according to current fitness level of the participant and medical approval is necessary [99]. Resistance exercise program should be started with a low level of intensity and the increase of intensity should be progressive.

The patient has to follow the prescription of the load’s weight, the technique and the number of repetitions indicated by the exercise specialist. In particular, at the beginning of the program, survivors should receive specific instructions by the fitness trainer, then they should be encouraged to exercise on their own. This kind of training can be safely performed two to three times a week of non-consecutive days [101].

The necessity of wearing compression garments during exercise is still uncertain: NLN recommends it especially for breast cancer survivors who have lymphedema, while Nelson, in his review, focused on a trial not requiring garments and pointed up that no changes to lymphedema status were reported among participants not wearing the compression garments [101, 102]. Summarizing, lymphedema is a collection of lymphatic fluid that commonly results from the removal of lymph nodes during the surgical treatment of cancer (in most of the cases of breast cancer). Exercise is a functional preventive and therapeutic strategy to manage lymphedema because it increases lymphatic fluid flow from the swollen areas.

## PSYCHO-PHYSICAL ASPECTS

### Pain

Cancer pain is a common symptom that negatively affects quality of life and significantly impacts on daily activities of cancer patients. It can be directly caused by cancer (due to compression of surrounding structures by the cancer mass or invasion of bone by metastatic tissue), related to the cancer consequences (such as bed sores due to stay in bed), induced by cancer treatments or caused/aggravated by comorbidities of the patient [103].

Cancer pain can be classified in acute, chronic (if it lasts more than 3 months) and breakthrough pain, that is an acute exacerbation of pain on the background of a well-controlled, persistent, stable cancer pain [104]. Cancer pain can also be distinguished in nociceptive or neuropathic. Nociceptive pain is due to the activation of nociceptors and can arise from somatic or visceral structures. Somatic pain is described as well-localized, sharp and cramping pain while visceral pain is difficult to localize and is described as dull, aching, and diffuse pain. Conversely neuropathic pain is directly related to lesions or diseases affecting the somatosensory system such as nerve compression or infiltration by the tumour, producing a tingling or burning type of pain. Neuropathic pain is often accompanied by other symptoms such as paresthesia, hyperalgesia and allodinia localized in the painful area [105].

There are several scales to assess pain intensity such as the verbal rating scale (VRS, the patient has to choose one adjective from a list of words to describe pain intensity), the numerical rating scale (NRS, in which the patient has to indicate a number between 0 -no pain- and ten -the worst pain-) and the visual analogue scale (VAS, a 10-cm line on which the patient has to mark a 1 cm line to indicate pain intensity) [27].

#### What kind of cancer/therapy can cause pain?

It is estimated that about 25% of cancer patients suffer from pain at cancer diagnosis, about 33% during treatment and approximately 75% during advanced stages [106], while the prevalence of pain in cancer survivors is ranging from 26 to 54% [107].

The localization of cancer strongly influences the prevalence of cancer pain, that is the highest (70%) in patients suffering from head and neck cancer, whereas ranges from 50 to 60% in patients affected by gynaecological, gastrointestinal, lung, breast and urogenital cancer (Table 1) [108].

Both surgery, chemotherapy and radiation therapy can cause cancer pain. Surgery usually causes acute pain, but sometimes the patient suffers from phantom pain (due to the removal of a body part) or develops a chronic post-surgical pain syndrome. Chemotherapy can determine pain arising from the infusion site (because of venous spasm, chemical phlebitis or extravasation) but also from mucositis, painful ulceration or peripheral neuropathy. Radiotherapy may cause osteonecrosis or chronic post-radiotherapy pain syndrome [109].

#### What are possible treatments for cancer pain?

Cancer pain is underreported and undertreated and it’s estimated that more than 40% of patients under therapy receive insufficient treatment [110].

The pharmacologic treatment for cancer pain comprehends opioid and non-opioid analgesics and also adjuvants.

Non-opioid analgesics include paracetamol (a well tolerated drug with antipyretic and analgesic proprieties) and non-steroidal anti-inflammatory drugs (NSAIDs) such as ibuprofen and naproxen, which inhibit cyclooxygenase (COX) an enzyme that converses arachidonic acid to prostaglandins, mediators of inflammation. The main side effects of NSAIDs are gastrointestinal and renal impairments [111].

Opioid analgesics interact with opioid receptors and are divided in weak (such as codeine, tramadol and dihydrocodeine) and strong opioids (such as morphine, oxycodone, hydromorphone, fentanyl, methadone and tapentadol). Common side effects of opioid treatment are sedation, dizziness, nausea, vomiting, constipation, physical dependence, tolerance and respiratory depression [112]. To enhance the efficacy of opioid analgesics and to reduce their adverse effect is possible to apply the opioid rotation [113].

The European Society for Medical Oncology (ESMO) guidelines recommends oral analgesic drug as the first line therapy for cancer pain according to the severity of the symptom: in particular paracetamol and/or NSAIDs are recommended for treating mild pain (defined as <3 on NRS); association of weak opioids and non-opioid analgesics for the treatment of mild to moderate pain (defined as 3-6 on NRS); strong opioids in combination with non-opioids analgesics for moderate–severe cancer-related pain (defined as >6 on NRS) [114].

An alternative pharmacological strategy for the management of mild to moderate pain is to administer low dose of strong opioids instead of the association between weak opioids and non-opioid analgesics [115].

Additional medications called adjuvants can be used to increase the effect of primary analgesic or to manage particular situations such as neuropathic pain and cancer related bone pain. Antidepressants (tricyclic antidepressants, SNRI) and anticonvulsants (gabapentin and pregabalin) are used to treat neuropathic pain, while bisphosphonates plays an important role in the management of cancer related bone pain [103].

A multidisciplinary approach to cancer pain includes psychosocial interventions (such as cognitive-behavioural treatment, relaxation training and stress management), which are able to impact on pain intensity and on and pain interference [116]. However, the pharmacologic therapy remains the most widely used approach for the treatment of cancer pain.

Cancer pain produces an important economic burden. Direct pain-related costs are related to analgesic medication and hospitalization for uncontrolled cancer pain. Cancer pain causes also indirect costs due to loss of productivity (the patient stops working, reduces his/her volunteer activities, leisure and housekeeping activities) and psychosocial costs (pain forces undesirable changes in lifestyle such as isolation) [117]. Also for these reasons is necessary to treat adequately cancer pain and to search new strategies to manage cancer pain.

#### Positive effect of physical activity on cancer pain

Despite the widespread conviction that patients suffering from cancer pain have to avoid physical exercise, a study on breast cancer patients suffering from muscle and joint pain during adjuvant chemotherapy found that patient pain perception was not worsened by training [118]. In support of these data, a study that proposed a resistance exercise program appropriately designed for prostate cancer patients with bone metastasis - which are at high risk for skeletal complications such as pathological fracture and bone pain - showed that supervised resistance exercise is safe and well tolerated [119].

Physical activity is a sustainable therapeutic alternative for chronic [120] and acute pain [121]; in fact physical exercise can provoke an elevation of pain thresholds [122].

The mechanisms by which physical exercise may work on pain are not completely known. Exercise is able to induce hypoalgesia (called Exercise Induced hypoalgesia, EIH), a relief from acute pain that characterizes the period after the activity and that may result from an endogenous pain inhibitory mechanism called conditioned pain modulation (CPM) [123] in which multiple analgesia systems (both opioid and non-opioid systems) are probably involved [121].

The EIH has been extensively studied in healthy individuals: results show that EIH is associated with higher-intensity and longer duration activities, but recently EIH has also been related to shorter bouts of moderate intensity [123]. Both acute isometric (static contraction with the manteinance of a defined joint angle), aerobic and dynamic resistance exercise types are able to reduce the perception of induced pain [124].

Different mechanisms have been described for the modulation of cancer pain by physical activity: it is able to reduce chronic muscle pain by lowering the phosphorylation, increased in chronic muscular pain, of a subunit (in particular NR1) of the N-methyl-D-aspartate receptor (NMDA, a receptor of glutamate) in the central nervous system [125].

The modulation of pain during and following exercise has also been related to Nitric oxide (NO), a soluble gas whose release is incremented during exercise. NO has several roles such as induction and maintenance of synaptic plasticity and homeostatic functions such as sleep control, appetite and body temperature. NO is also involved in central pain modulation, since high levels of NO are associated with increased release of GABA, a neurotransmitter involved in pain control. The exercise induced pain modulation is associated with the NO/cGMP/K_ATP_ pathway at both spinal, supraspinal and peripheral levels in mouse model [126, 127].

Another study, carried out in mouse model with peripheral nerve injury, found also an association between the analgesic effect of exercise and the central monoaminergic systems, reducing neuropathic pain by enhancing brainstem serotonin (5-HT) neurotransmission [128]. In mouse also the noradrenergic system has been described to be involved in exercise induced pain modulation. Physical exercise was associated with the activation of α2-adrenergic receptors (α2-ARs) increasing the pain threshold [129].

Another hypothesis to explain the modulation of pain induced by exercise is that it may influence macrophage phenotype, promoting M2 (which secrete anti-inflammatory cytokines) instead of M1 phenotype (which secrete pro-inflammatory cytokines) to increase the anti-inflammatory cytokine IL-10, that can lower nociceptors sensitization. The percentage of M2-macrophages and IL-10 were significantly increased in physically active mice and the block of IL-10 prevented the analgesic effect of exercise [130].

Even if physical activity seems to have an important role in modulating pain, the exercise effects on pain in cancer patients have been little studied. Several studies evaluated the effects of exercise on various types of cancer pain during and after cancer treatment, with different results.

One of the most studied exercise approach against cancer pain is physical activity targeting shoulder pain among breast cancer patients treated with surgery, chemotherapy, and radiation therapy: results show that exercise reduces shoulder pain related to breast cancer treatment [131].

A randomized controlled trial conducted on patients undergoing treatment for solid tumours suggests that a home-based aerobic exercise intervention (such as walking or cycling) significantly decreases the level of pain (Table 2) [132]. A study conducted on breast cancer survivors found that exercise has positive influence on breast or chest-wall pain [133]. A recent trial proposed an internet-based tele-exercise program (3 non consecutive session per week, approximately 90 minutes each day, divided into three sessions: warm-up, resistance and aerobic exercise training, and cool-down) for breast cancer survivors. After the intervention, the tele-rehabilitation group showed a significant decrease in pain severity and pain interference compared with the control group, which receives only a written recommendation for exercise, suggesting that a structured exercise supervised by physical trainers increases exercise adherence of cancer patients and survivors [134].

Evidence suggests positive effects of exercise in most of neoplastic patients suffering from cancer pain and they can take advantage of exercise approach. Nevertheless, the type of exercise indicated for each type of cancer pain has to be personalized.

### Fatigue

The National Comprehensive Cancer Network (NCCN, USA) defines Cancer-related fatigue (CRF) as a distressing, persistent, subjective sense of physical, emotional, and/or cognitive exhaustion related to cancer or cancer treatment that is not proportional to recent efforts and interferes with daily activities [135]. The tiredness that characterizes CRF is more intense and more severe than normal fatigue and is not reduced by sleep or rest [136]. Conversely, rest may exacerbate CRF.

The pathophysiology of fatigue is multifactorial and the specific mechanisms involved in it are unknown. Causes of fatigue may be referred to metabolic imbalance, immune system disorders and increased cytokines such as NF-κB and TNF-α (Table 1) [137]. The systemic inflammation that characterized cancer patients derives from both cancer and related treatments. In fact radiotherapy and chemotherapy may cause a massive release of inflammatory cytokines from necrotic tissues. Fatigue can also be related to anaemia and sleep disturbances [138]. About 70-80% of patients experience fatigue during treatments, but approximately one third of survivors still suffer fatigue after the end of the therapy: this is called persistent CRF and has a significant impact on the social and economic life of people affected by this problem [139].

#### What kind of cancer/therapy can cause fatigue?

Any kind of cancer can cause CRF, but fatigue occurs more often with malignant solid tumours such as pancreatic and breast cancer, and hematologic disease as lymphoma [137]. Fatigue is experienced by 80% of individuals who receive chemotherapy and/or radiotherapy, nevertheless also surgery and hormonal therapies can cause CRF. For people undergoing chemotherapy, CRF follows the cycle of chemotherapy: it increases in the first few days and then it diminishes during the interval between two treatments, while for those patients who receive radiotherapy, fatigue usually gets worse as the treatment goes on.

#### Which are the possible treatments for fatigue?

Fatigue in patients with cancer has been under-reported and under-treated. The patient doesn’t declare this symptom because usually thinks that nothing can be done to improve his/her condition. This is why fatigue should be screened, classified and the physician has to inform the patient about fatigue and its natural history. This education should be offered to all patients with cancer, especially to those who are at the beginning of fatigue-inducing treatments [140].

Many interventions have been evaluated to prevent or treat CRF. The treatment of CRF can be pharmacologic or non-pharmacologic. Pharmacologic interventions include stimulants (such as methylphenidate, a central nervous system stimulant similar to amphetamine and modafinil, a weak dopamine reuptake inhibitor used for the treatment of narcolepsy), antidepressants (in particular selective serotonin re-uptake inhibitors - SSRIs - like sertraline) [141] and steroids such as dexamethasone [142].

Non-pharmacologic interventions include: psychosocial interventions such as support groups [143], comprehensive coping strategies [144] and psycho-educational therapies [145], mind-body interventions like mindfulness-based approaches [146], acupuncture [147] and physical activity.

#### Positive effect of physical activity on fatigue

Physical activity is one of the non-pharmacologic interventions that have the strongest evidence for treating fatigue. A large meta-analysis of 70 studies, 27 of which included patients after treatment, reported that exercise significantly reduces CRF by 32% or 38% during or after cancer treatment respectively [148].

A very recent meta-analysis of 113 randomized clinical trials (11525 patients) compared the mean weighted effect sizes (WESs) of pharmaceutical treatment, psychological strategy, exercise and exercise plus psychological interventions on fatigue. The authors found that exercise has the greatest impact in reducing fatigue when compared to other therapies [149]. According to this evidence, it is recommendable to encourage all patients to try a moderate level of physical activity during and after cancer treatment.

The mechanisms through which exercise reduces fatigue have not been fully elucidated. It has been suggested that physical activity could reduce CRF by improving functional capacity, resulting in a reduced effort to sustain daily activities and improvement in the capacity to recognize a reduced body weakness and exhaustion [150]. Resistance, aerobic, flexibility and mixed training programs all demonstrated to improve CRF, but it is still under debate which form of exercise is superior to others.

ASCO recommends to patients 150 minutes per week of moderate aerobic exercise with an additional two to three sessions of strength training, such as weight lifting, unless contraindicated [140]. The exercise specialist has to develop an exercise program based on the patient’s age, gender, type of cancer, complications, comorbidities and physical fitness level.

Previous guidelines suggested that exercise prescriptions depend on the level of fatigue. In fact, asking patients to rank fatigue on a numeric rating scale from 0 to 10, CRF can be classified in mild (1-3), moderate (4-6) and severe (7-10). Patients with mild fatigue had to perform progressive aerobic exercise program that includes 20–30 min per session, 3 to 5 days per week, at 60%–80% of maximum heart rate and resistance exercises that included 8–10 exercises for major muscle groups of upper and lower extremities and trunk, 2 to 3 days per week. Patient with moderate fatigue were encouraged to increase exercise through repeated bouts of 5–10 min per session; the resistance exercise program was similar to the aforementioned one for patients with mild fatigue, but for patient with moderate fatigue it was proposed to increase exercise frequency or duration before increasing intensity. Patients with severe fatigue were encouraged to try frequent sessions of low-intensity exercise (e.g. walking/biking for 5 to 10 minutes) spaced throughout the day (Table 2) [151].

Complementary therapies, such as yoga and muscle relaxation have been tried alone or in combination with physical exercise [135]. Yoga is a mind-body practice that combines physical exercise such as stretching and specific body postures with deep relaxation and meditation accompanied by breath control [139]. A randomized control trial study conducted on breast cancer patients who had completed cancer treatments determined the efficacy of yoga intervention in breast cancer survivors with persistent post-treatment fatigue [152].

In summary, physical exercise alone or in association with alternative approaches such as Yoga, showed positive results in patients with cancer related fatigue, so exercise may be a feasible, safety and effective strategy to manage CRF.

### Sleep disorders

The American Academy of Sleep Medicine defines five major categories of sleep disorders: insomnias, sleep apnea, hypersomnias, circadian rhythm sleep disorders and parasomnias. Cancer patients and cancer survivors often suffer from insomnia, which is defined as the difficulty of initiating and maintaining sleep and/or poor sleep quality. The diagnostic criteria for cancer related insomnia are: frequency of at least 3 nights per week, delayed sleep onset (>30 minutes to initiate sleep), difficulty with sleep maintenance (>30 minutes nocturnal waking) and significant impairment in daytime function [153]. The prevalence of insomnia symptoms in cancer survivors ranges from 18 to 68% depending on the cancer type [154].

Sleep disturbances can be perpetuated and insomnia often becomes chronic, persisting for at least one month and causing several form of daytime impairment [155]. Chronic insomnia causes physical effects such as fatigue, cognitive dysfunction and headache, psychological consequences such as depression and increased risk of suicide, as well as psychosocial problems such as lost work productivity and poor relationships [156].

The pathophysiology of sleep disorders is not clear but many predisposing factors can determine insomnia among cancer patients since they suffer from anxiety and depression (conditions that are often associated with sleep problems also in healthy people) and since they are exposed to cancer treatments and supportive care, which may contribute to sleep disturbances [157]. Older age, female gender, familiar and personal history are non-modifiable factors that may also contribute to cancer related insomnia [158].

#### What kind of cancer/therapy can cause sleep disorders?

Cancer treatments may influence insomnia: the prevalence of sleep disturbances in cancer patients who undergo surgery, radiation therapy or chemotherapy is 49%, 39% and 35% respectively [159].

Lung (34%), breast (48%) and gynaecological (30%) cancer patients have an higher sleep problem incidence and experience sleep disturbance. Hypnotics and sleep medication are used by 40% of lung cancer patient and by 28% of breast cancer patients. Prostate cancer patients suffer from sleep maintenance due to frequent voiding. Gastrointestinal patients show the lowest prevalence of sleep disorders (7%) (Table 1) [160].

#### What are possible treatments for sleep disorders?

Treatment of sleep disturbances is multimodal and includes both pharmacologic and non-pharmacologic approaches. Pharmacologic strategies include hypnotics (both benzodiazepine and non-benzodiazepine such as zopiclone and zolpidem), antidepressants, antihistamines and melatonin. The first line pharmacotherapy approach is the combination of short-acting benzodiazepines with non-benzodiazepines hypnotics for less than 4 weeks, but in most cases is necessary to continue therapy for long periods [160], even if it’s associated with side effects such as dependence and discontinuation syndrome. Tricyclic antidepressants (such as amitriptyline) and trazodone (an antidepressant of the serotonin antagonist and reuptake inhibitor class) are used to improve sleep continuity [161]. Melatonin seems to promote sleep by regulation of the sleep–wake cycle and is associated with an improvement in subjective sleep quality [162].

Non-pharmacologic therapies comprehend relaxation therapy, sleep education, stimulus control, sleep restriction and cognitive behavioural therapy. Relaxation therapy reduces somatic and muscular tension by first slowly tensing and then relaxing each muscle group and is associated to meditation to lower thought and preoccupations. Sleep education is used to remove environmental disturbing factors and discourage behaviours that may interfere with sleep. Stimulus control is important to consolidate the association of the bed/bedroom with sleep (for example it is recommended to not watch TV or read in bed) and to reinforce the sleep-wake cycle (going to bed at the same time every night and waking up at the same time every day). Sleep restriction therapy consists in reducing the time for sleeping, to improve sleep quality [163]. Cognitive behavioural therapy combines different approaches using some of the above mentioned techniques and is able to improve sleep efficiency with a durable effect, with positive consequences on mood [164].

#### Positive effect of physical activity on sleep disorders

Exercise has many effects on sleep duration, architecture and quality.

Exercise directly influences the circadian system, resulting in an increased total sleep time as well as time in deep sleep (slow-wave sleep), which improves sleep quality. In fact, deep stages of non-rapid eye movements (REM) sleep induce neurophysiological restoration and a physiological muscle and tissue recovery. In addition, exercise reduces sleep-onset latency (SOL, the transition from full wakefulness to sleep) and REM sleep [165].

The direct effect of decreased REM sleep on sleep quality remains unclear, but it is suggested that REM sleep reduction has an antidepressant effect, so exercise acts also indirectly by reducing depression, a predisposing factor of sleep disturbances [166].

Exercise also influences thermogenic regulation, causing temperature elevation. The increase in core temperature seems to promote the initiation of sleep by activating heat dissipation mechanisms controlled by the hypothalamus. In addition, people affected by insomnia have an impaired nocturnal temperature down-regulation and the thermogenic effects of exercise may regulate this mechanism [167].

Furthermore, exercise may modulate sleep-mediating cytokines, such as IL-6 and TNF-α. In particular, exercise promotes the release of IL-6 in direct proportion to exercise intensity, muscle mass involved during exercise, duration and training status. The peculiarity of exercise-induced release of IL-6 is that the release is immediate but transient. This time course of IL-6 has also an anti-inflammatory action: promotes the release of soluble TNF-α receptor (sTNF-R) and inhibits the production of TNF-α. IL-6 and TNF-α are elevated in cancer patients and are able to activate the hypothalamic-pituitary-adrenal (HPA) axis resulting in short sleep duration and sleep disturbances [168].

The USA National Sleep Foundation considers exercise as a non-pharmacological intervention to improve sleep quality. Many studies focused on the exercise approach to treat sleep disturbances showed significant improvements in objective (Polysomnography) and subjective (sleep quality scales) measurements of sleep quality among patients with chronic primary insomnia [169]. These benefits were also confirmed upon increased serotonin release and activity that are promoted by acute exercise in middle aged and older adults with sleep disturbances [170, 171].

When exercise should be performed to improve sleep quality? Morning exercisers get the most favourable sleep outcomes. However, despite the belief that exercise should not be performed in the evening because it may reduce sleep duration and quality, data reports that individuals who performed vigorous evening exercise obtained an equal or higher sleep quality compared to the days without exercise [172]. Sleep ameliorations after chronic exercise are thought to be similar to improvements after hypnotic drug use [173]. When compared to hypnotic agents, regular exercise training has similar results in improving sleep quality, reducing SOL, number of awakenings and wake after sleep onset (-WASO-: the amount of time a person spends awake after falling asleep) [167]. A recent systematic review of three previous meta-analysis concluded that exercise improves sleep outcomes, such as overall sleep quality, subjective sleep and sleep latency [174].

Several randomized control trials also explored the possible benefit of exercise as a therapy for sleep disturbance in cancer patients, with different results [175, 176]. Courneya et al. conducted a trial about the effect of exercise on sleep quality in lymphoma patients and they found that aerobic exercise significantly improved sleep quality in obese patients and patients that underwent chemotherapy (clinical features that increase the risk of poor sleep quality) [177].

Which kind of physical exercise could reduce sleep disorders? Long-term moderate aerobic exercise (6-month exercise intervention, three days a week, 50 minutes of treadmill continuous session) showed significant improvements in sleep in individuals with chronic primary insomnia [178]. Most intervention adopted by trials concerning the effect of exercise in cancer patients were home-based aerobic walking programs. Tang et al. carried out a trial concerning the effects of a home-based exercise intervention on sleep quality in cancer patients. The home-based exercise consisted in walking briskly 3 days a week, for 30 min a day for a period of 8 weeks, and the results reported significantly improvements in sleep quality [179]. Cheville et al. proposed a home-based walking and a strength training for 8 weeks to patients with stage IV lung and colorectal cancer and found benefits in sleep quality. The strength training consist in Rapid, Easy, Strength Training (REST) exercise program, which included two sets of five exercise, one for the upper and one for the lower body. Each REST exercise has to be repeated 10 times, twice a week, for a total of four sessions, two for upper and two for lower body [180]. A recent meta-analysis concludes that walking is an exercise effectively improving sleep and suggests that walking exercise program should be included in a multimodal approach to manage sleep disorders in people with cancer (Table 2) [181].

Also relaxation exercises are able to improve sleep quality by reducing muscle tension and promoting muscle relaxation [163]. In particular, Yoga is a well-tolerated type of exercise with a potential role in ameliorating insomnia symptoms among cancer patients. Doing breathing and meditation exercises ranging from one to five sessions/week for 50-120 minutes per session for 4-26 weeks leads to reduction in insomnia symptoms and improve sleep quality [182]. A randomized controlled trial found that a yearlong aerobic and stretching exercise program both improved sleep quality and reduced the use of sleep medications [183]. In conclusion, both aerobic exercise (in particular walking program) and relaxation technique (such as Yoga) can be considered alternative treatments for insomnia in cancer patients by increasing total sleep time, improving sleep quality, influencing thermogenic regulation and modulating sleep-mediating cytokines.

## PSYCHOLOGICAL ASPECTS

### Depression, anxiety

According to the Diagnostic and Statistical Manual of Mental Disorders, Fifth Edition (DSM5), the diagnosis of anxiety requires excessive anxiety or worries that are difficult to control and impair function, along with at least 3 of the following symptoms lasting most of the time over the past 6 months: restlessness or feeling keyed up or on edge, being easily fatigued, difficulty in concentrating, irritability, muscle tension and sleep disruptions. A major depression diagnosis requires at least 5 of the following symptoms most of the time in the past 2 weeks: sadness (feeling hopeless, empty, or depressed), loss of interest or pleasure in most activities, significant changes in weight or appetite, sleep disruptions (including sleeping too much or too little), psychomotor slowing or agitation that is observable by others, fatigue or loss of energy, feelings of guilt or worthlessness or feeling like a burden, difficulty concentrating or indecisiveness and thoughts of being better off dead or active suicidal thoughts or plans [184].

Depression and anxiety are two common symptoms in cancer patients and survivors. The prevalence of depression in cancer patients ranges from 8% to 24% depending on the type of instrument used to assess the diagnosis of depression (either diagnostic interviews or self-report instruments), type of cancer and treatment phase [185]. Incidence of depression (assessed by diagnostic interview) is 11% in patients with breast cancer or head and neck cancer, 3% in patients with lung cancer, 8% in patients suffering from haematological malignancies and 23% in patients with gynaecological cancer. Prevalence of depression is highest during cancer treatments and then decreases after the end of the therapies, affecting 9% of cancer survivors (Table 1) [186]. In long-term cancer survivors the prevalence of anxiety symptoms in survivor is higher, ranging from 18% to 20% (especially for those who have a diagnosis of lung cancer or melanoma) [187].

Cancer patients might suffer from depression and anxiety for many reasons: in response to cancer diagnosis, to the symptoms associated with cancer and to side effects of cancer therapies (such as pain and fatigue) and because of fear of disease recurrence or progression [188]. In fact, after the end of cancer treatment, survivors (especially those who are regularly scheduled testing for recurrence) experience the fear of recurrence (FOR), so they are hypervigilant and worried about physical sensations. FOR can promote anxiety and symptoms of posttraumatic stress [187].

Depression in patients with cancer may cause increased hospital stays, physical distress, lower treatment adherence, reduced quality of life [189] and increased risk of suicide [190]. Patients with advanced cancer disease experience major depression, due to an increased number and severity of symptoms and a reduced life expectancy as the disease progresses [191].

#### What are possible treatments for depression and anxiety?

Current treatments for depression in cancer patients and survivors include pharmacological interventions and psychotherapy, even if a high number of cancer patients with depression do not receive a proper antidepressant treatment [192]. Pharmacologic treatment for depression in cancer patients envisages tricyclic antidepressant (TCAs), monoamine oxidase inhibitors (MAOIs), SSRIs, SNRIs and other agents, such as mirtazapine and bupropion.

In cancer patients there are low quality evidences about the effectiveness of antidepressants for the prevention and treatment of depressive symptoms [193]. In addition, antidepressants are contraindicated for some cancer patients, for example SSRIs may be contraindicated for those patients undergoing certain anti-hormonal therapies, because they may lower levels of tamoxifen [194].

Pharmacologic treatment for anxiety in cancer patients includes antidepressants (such as imipramine and mirtazapine) and benzodiazepines (such as alprazolam and lorazepam) [195].

Psychosocial interventions can reduce depressive and anxiety symptoms in patients with cancer. Some of the most common interventions are: cognitive behavioural therapy, supportive-expressive group therapy (SEGT, which encourages the expression of feelings and fears regarding the life threatening illness and increases social support), problem solving therapy (a psychological intervention that helps the patient to identify and approach the problems of its life) and mindfulness exercises (a relaxation training to reduce stress and concerns) [195, 196].

#### Positive effect of physical activity on depression and anxiety

Exercise is a prevention strategy that reduces the onset of clinical depression and anxiety [197]. Physical activity is associated with decreased symptoms of depression, increased life satisfaction and cognitive functioning and promotes a mental well-being [198]. Exercise has also an anxiolytic effects on anxiety disorders. People who perform regular physical activity have a reduced prevalence of panic attacks, social anxiety disorders, specific phobia such as agoraphobia compared to those who do not [199].

The neurophysiologic mechanisms potentially responsible of these anxiolytic and antidepressant effects are not known, but several hypotheses have been formulated.

Depression is associated with structural changes of central nervous system, such as a reduction of volume of the hippocampus, detected by imaging studies [200]. Brain neurogenesis is probably increased by exercise, which could influence hippocampal neurogenesis through promoting the release of beta-endorphins, VEGF, BDNF and serotonin [201]. Exercise is also associated with increased levels of endocannabinoids, which have an anxiolytic effect and promote a sense of well being [202].

The hypothalamo-pituitary adrenal axis is influenced by physical exercise exerting the ability to improve mood (in particular increases the release of adrenocorticotropic hormone, ACTH and decreases the cortisol production) [198].

Physical activity is capable to lower anxiety sensitivity (a characteristic inclination to think that anxiety itself will lead to catastrophic consequences) [203]. In addition, physical exercise increases the physiological resistance to stressful stimuli and lowers negative psychological states, by different changes in neuroendocrine regulation, such as increases in body temperature, modulation of the central serotonergic systems and endorphin production. Exercise is also able to reduce anxiety and depression because it increases the quality of sleep and lowers sleep disturbances. Furthermore, through physical activity patients develop an increased sense of mastery, the sensation of controlling and determining his/her own life and expectations. An increased sense of mastery promotes positive psychological states and helps to restore hope [204]. Exercise causes also psychological benefits such as reduction of negative emotions such as anger, contempt, disgust, guilt, fear and nervousness (sensations that characterize the negative affect) lasting several hours after the end of exercise.

Exercise has also long-lasting effects (that are produced when exercise becomes part of the routine) such as improvements in global self-esteem as well as in physical self-concept [205].

The guidelines of the American Psychiatric Association (APA) recognize that exercise has a role in the treatment of depression by improving mood and consider exercise as a non-pharmacological intervention – alone or in addition to pharmacologic treatment – to manage depression. A meta-analysis of 49 trials that compared exercise with no treatments or alternative treatments for anxiety found that the exercise group showed statistically significant anxiety reductions when compared to control and to alternative treatment groups (except for pharmacotherapy) [206]. A study that compared physical exercise (supervised or home based) to sertraline (a SSRI) for 4 months found that exercise was as effective as drug administration in lowering depression symptoms [207]. After one year of follow up, the group of patients that continued exercise maintained the benefits of physical activity. Nevertheless, the group of patients that received sertraline and that started to exercise after the end of the original study showed a depression amelioration, suggesting that exercise maintains his positive effect on depression for a long time (more than one year) and that if exercise is combined with antidepressant, it may increase the effect of the medications [208].

Another study that focused on the comparison between physical exercise (running on a treadmill for 30 minutes 3 times a week for 16 weeks) and sertraline found that at month ten of follow up the exercise group showed a significant reduction of depression when compared with the sertraline group. These data suggest that exercise may have a more lasting effect than medication [209].

Many randomized controlled trials found a positive impact of exercise on depression and anxiety symptoms, both on lung, colon and breast cancer patients [210–213]. The anxiolytic and antidepressant effects of physical activity has been demonstrated in both patients undergoing treatment [214, 215] and cancer survivors [216].

#### Which kind of physical exercise could reduce depression and anxiety?

Several studies and metanalisis concerning both cancer patients and survivors tried to find which type and dose of exercise can improve the symptoms of depression or anxiety. A meta-analysis done by Brown et al. [217] that included 40 exercise interventions on cancer survivors found that the reduction of depressive symptom expanded as the weekly volume of aerobic exercise increased (Table 2). These results were confirmed by another metanalysis [218] that found larger effects of exercise programs that were supervised or partially supervised (not performed at home) and that lasted at least 30 minutes. An high exercise frequency (five times per week) of at least 30 minutes produced larger effect than a low exercise frequency (two to four days for week) and exercise programs that lasted from 10 to 16 weeks were reported to be more effective than programs that lasted less than 9 weeks. [219]. Courneya et al. [189] compared different exercise doses and types on psychosocial distress in breast cancer patients undergoing chemotherapy and confirmed that higher volumes of exercise were effective in patients with clinical levels of depressive symptoms at baseline.

A study conducted on lung cancer patients proposed a home-based walking exercise (moderate-intensity walking-exercise programme consisting of 40 minutes per session, 3 sessions per week for 12 weeks) and found that this kind of training can reduce anxiety and depression [210].

In summary, positive effect of exercise on anxiety and depression is well evidenced and exercise is a potentially powerful treatment of depression and anxiety.

### Quality of life and self esteem

Quality of life is a broad multidimensional concept referred to subjective evaluations of physical, emotional and social dimensions, as well as stress level, sexual function and self-perceived health status. Health-related quality of life (HRQOL) focuses on the influence that physical and mental health status directly causes on quality of life. HRQOL is usually measured through multiple questions regarding self-perceived health conditions and physical and emotional functioning [220].

The cancer diagnosis and its treatment change forever patient’s life and affect his/her quality of life, according to physical symptoms and psychological aspects such as the perception of decreased life expectancy. As the number of cancer survivors continues to increase, there is an increasing focus on long-term management of quality of life in these patients.

Global self esteem is generally defined as the individual’s evaluation of his/her own worth, but is also conceptualized as the sum of domain-specific self-concepts (such as physical self-concept) [221]. Physical self-concept is a person’s perception or description about the body, appearance and physical abilities. Cancer patients and survivors experience negative changes in their self-images (such as hair loss, weight changes and surgery scars) and self-concepts. Consequently, it’s difficult for cancer patients to find a psychological balance in response to the changes that characterized the cancer diagnosis and treatment. The adaptation or the psychosocial adjustment to cancer is a process in which people try to deal with their suffering and requires a constant attempt to take control over events triggered by the disease. People with high self-esteem trust in their capacity to deal with challenges and difficult situations and have greater chances to react to the disease and to face the antineoplastic treatment [222] while cancer patients with low self-esteem have higher levels of anxiety and depression and report a worse quality of life (also for social/family and emotional aspects) [223].

#### Does physical activity influence quality of life in cancer survivors?

Obesity and inactivity are related with poorer quality of life among cancer survivors [224, 225], while physical activity is able to improve HRQOL both in healthy individuals [226] and in patient affected by chronic and invalidating diseases [227].

Several trial and reviews concerning different kind of cancer focused on the positive impact of exercise on quality of life. Koutoukidis et al. found that physical activity improved quality of life of endometrial cancer survivors [228]. In head and neck cancer patients, which experienced an important decrease of quality of life according to the impaired ability of swallowing, breathing and speaking, it was found that exercise may play an important role to increase their quality of life (Table 1) [229].

A systematic review concluded that exercise positively influenced quality of life in breast cancer patients [230] and Philips et al. found that moderate-to-vigorous physical activity was associated with improvement in many HRQOL indicators also in breast cancer survivors [231].

These results are confirmed by a Cochrane review that included 40 trials (with 3694 participants) and evaluated the effectiveness of exercise on HRQOL in cancer survivors. Participants were diagnosed for breast, colorectal, head and neck, lymphoma and other cancers. The exercise intervention could be variable and included strength training, resistance training, walking, cycling, yoga, Qigong, or Tai Chi. The results suggested that exercise compared with control has a positive influence on physical, psychological and social aspect of HRQOL. Evidence indicates that since exercise increases physical functioning, reduces fatigue syndrome and sleep disturbances is capable to improve quality of life. Exercise influences psychological well being by lowering the person’s worries about his/her cancer and improving the body image/self-esteem. Exercise also helps the way the person deals with emotions and sexuality and has a positive impact on social well-being.

However, physicians do not recommend exercise with adequate incentive, push and motivation, even if it has been found that patients who received oncologists’ exercise recommendation and motivation significantly increased moderate intensity exercise and exercise frequency (Table 2) [232].

Quality of life depends on various aspects and different types of exercises may have an influence on different aspects of quality of life. An interesting example of non-conventional physical activity in survivors was investigated by a Danish study that tested a community-based recreational football as a novel strategy to promote physical activity in prostate cancer survivors, with positive results [233].

A systematic review concerning breast cancer survivors indicated that a combination of both aerobic exercise and strength training could provide the most benefit for the quality of life [230]. More data are needed to determinate the optimal exercise frequency and intensity based on the type of cancer.

#### Does physical activity influence self-esteem in cancer patients and survivors?

Physical exercise (both aerobic, weight and relaxation training) is an effective strategy to improve physical self-concept and global self-esteem. Exercise acts on physical self-concept by increasing the level of endurance, the capacity to learn sport skills and maintain physical fitness. Physical activity also improves the perceived attractiveness of the body and self-confidence in appearance, as well as the perceived physical strength (muscular strength, muscle tone and self-confidence in physical activities requiring strength) [205]. Exercise training also enhances the sense of achievement related to perseverance, resolution and tenacity in performing the program [234].

There is consistent evidence that physical activity is associated with improvement in self esteem in breast cancer survivors [235]. A recent study showed that breast cancer survivors with higher levels of physical activity obtained greater self-efficacy. Self-efficacy is the awareness of being able to dominate specific activities and situations or to achieve intended results. Breast cancer survivors with greater self-efficacy reported higher levels of physical self-worth and higher physical self-worth was associated with greater global self-esteem [236].

Physical self-esteem consists of three subdomains: physical condition, attractive body and physical strength. Resistance exercise training seems to be the most effective type of training to improve self-esteem in breast cancer survivors because it acts on all the three subdomains of physical self-esteem, while aerobic training apparently improved mostly the attractive body domain. The combination of the two programmes (resistance and aerobic) is not more effective than the single-modality programs (Table 2) [237]. In conclusion, there is a growing body of evidence indicating that physical activity (especially resistance training) results in increased self esteem on cancer patients.

## ACCESSIBILITY TO PHYSICAL ACTIVITY TREATMENT

### Barrier to physical activity treatment

Cancer survivors are not easy to convince in participating physical activities programs, in fact they often reports barriers to physical exercise. Barriers can be classified in disease-specific limitations, such as illness, pain, fatigue; socio-economic barriers such as financial difficulties, lack of time, transportation issues, child care problems [138] and individual barriers such as anhedonia, lowliness, social withdrawal, decreased self-confidence, lack of interest/motivation and the perception of being “too busy” or having “no willpower” [238]. The most common barriers are the fear of pain or fatigue because of the movements and the belief that cancer management requires rest rather than exercise. Although the presence of these barriers, about 50% of cancer survivors declared they would be interested and feel capable to exercise [234].

### Strategies to make exercise a more accessible therapy

#### Motivation: start moving!

To overcome these barriers survivors should be encouraged by counselling and motivational interviewing with physicians, physical therapists and trainers. At the beginning of the program patients should explain their expectations and attitudes toward physical exercise. Then survivors should receive an education on exercise training principles from physical therapists and trainer and at least the first sessions should be supervised to ensure correct exercise performance. The role of the physical trainer appears fundamental in increasing exercise adherence of cancer patients and survivors to the program. Therefore in each program there is the need of educated practitioners to work in team with physicians and physical therapists.

Survivors should be encouraged to monitor both the physical activity (e.g., heart rate monitor, perceived exertion) and the daily activities outside the exercise setting and to report the perception of symptoms before, during, and after exercise. This progressive education of the patient provided by the physical therapists and trainer may be the key to keep the patient’s adherence to exercise program. It’s important to encourage social contact during exercise: exercise with a partner or in a group setting [151].

#### Time to exercise!

Ideally, exercise should begin at the time of diagnosis, to reduce the cancer symptoms and the progressive side effects of the treatments. If physical activity is offered at the beginning of the disease, patient probably has the physical capacity to undertake and complete programmes, and will get satisfaction from physical activity, will get used to it and will maintain an active physical activity during and after cancer treatments [57].

#### Motivation: keep moving!

Only about 50% of patients complete the exercise programme [239]. Patients’ adherence to exercise is a complex phenomenon related not only to the physical aspects (such as patient condition and treatment side effects) but also to psychological, motivational and behavioural factors. Adherence to exercise programs can influence treatment outcome and has to be sustained by different approaches. In particular, the “exercise stage of change” of cancer patients and the intention to operate a health-changing behaviour are significant predictor of exercise adherence [240]. The exercise stages of change derive from the transtheoretical model of behaviour change (TTM, a widespread model to explain personal behavioural change) applied to regular exercise and analyses change as a progression through six different stages [241].

Motivation may increase as the physical training goes on. A recent study proposed to rectal cancer patients to attend supervised aerobic exercise sessions during and at the end of neoadjuvant chemo-radiotherapy treatments. Patients were provided with the option of continuing with the supervised exercise program, completing an unsupervised exercise program, or a combination. The study compared perceived benefits, harms, enjoyment, support, difficulties and barriers for exercise both during and after neoadjuvant chemo-radiotherapy treatment. There were statistically significant improvements in perceived enjoyment, difficulties and support during the supervised exercise program: this positive motivational response influenced exercise adherence. In fact, after the end of neoadjuvant chemo-radiotherapy treatments, patients reported being quite motivated to start with the unsupervised exercise program until the surgical treatment. Surprisingly, even if the exercise was not supervised, patients reported an increased perceived social support. The majority of patients also reported that the exercise program post neoadjuvant treatment helped them to prepare for surgery [242].

A study conducted on lung cancer survivors found that a 10-week supervised progressive resistance training program may improve some motivational outcomes on the basis of the theory of planned behaviour (TPB, according to which intention is the principal determinant of behaviour). In particular there was a significant increase in self-efficacy (the ease of performing a behaviour) and the perceived controllability (the control the individual has over performing the behaviour) [243].

The HELP (Healthy Exercise for Lymphoma Patients) trial reported that a 12-week one-to-one supervised aerobic exercise program in lymphoma patients receiving chemotherapy had positive effects on motivation, resulting in significant improvements in exercise behaviour at 6-month follow-up [244].

In summary, patients’ adherence to exercise increases as patient keep moving, although initially his/her motivation may be poor and is more strict if a supervision runs along the program.

#### Social contact and support

The possibility to perform physical activity depends on environmental factors such as social support and positive social relationships that make easier to exercise. In fact, receiving support from family and friends as well as from the community predicts engagement in healthy behaviour such as physical activity [245, 246]. In particular, caregivers play an important role: their social support in the form of companionship (being physically active together with the cancer patient or assisting the patient in performing exercise), motivation, or health promotion facilitates cancer patients to exercise by providing encouragement. Consequently, educating caregivers (as well as cancer patients and survivors) on the importance of performing physical activity may be an effective strategy to increase participation to physical activity [247]. Anyway, caregivers should be educated to avoid social control behaviours, such as criticizing the patient for his or her insufficient physical activity and nagging the patient to exercise more, because this “pressure” can exacerbate a “boomerang” effect called reactance (a perception according to which behavioural freedoms are threatened) which results in detrimental effects (reduction or stop of exercise) [248].

#### Patient preferences

Several studies assessed cancer patients express preferences by submitting them questionnaires. In these studies the majority of patients showed preferences for walking and strengthening exercises, chose the morning activities and moderate intensity exercises. Patients also reported that the better time to start physical activity was before starting treatment or 3–6 months post-treatment. Motivational strategies such as exercise being fun, incorporating music and variety, establishing goal setting were appreciated by the patients and would facilitate them to exercise [234, 249]. Anyway, the patients opinions remain various, so the most appropriate strategy to obtain a widespread participation in exercise programs would be to offer a multidisciplinary approach with the possibility to choose different types of activities according to patients’ symptoms and disability and different timing and settings, which allow patients to exercise in group or at home.

The evidences that we reported show that physical exercise provides benefits on several symptoms of cancer and side effects derived from cancer treatments regarding physical, psychophysical and psychological aspects. Thus, exercise in cancer patients and survivors should be proposed and started as soon as possible to enhance motivation. A multidisciplinary offer of training, frequency and setting of physical activity as well as the caregiver’s support are important to increase exercise adherence. Finally, the most effective type of exercise, optimal exercise frequency and intensity based on the type of cancer should be prescribed and supervised as a therapeutic program like it happens for the type, dose and duration of a drug treatment.
